# Vaccines to prevent leishmaniasis

**DOI:** 10.1038/cti.2014.4

**Published:** 2014-03-14

**Authors:** Rajiv Kumar, Christian Engwerda

**Affiliations:** 1 Immunology and Infection Laboratory, QIMR Berghofer, Brisbane, Queensland, Australia

**Keywords:** immunity, leishmania, parasites, vaccines

## Abstract

Leishmaniasis is a parasitic disease that encompasses a range of clinical manifestations affecting people in tropical and subtropical regions of the world. Epidemiological and experimental data indicate that protection from disease can be achieved in most people. In addition, we know how the host immune system must respond to infection in order to control parasite growth. However, there is still no vaccine for use in humans. Here, we review our understanding of host immunity following *Leishmania* infection and also discuss recent advances in the development of vaccines to prevent leishmaniasis, highlighting a new promising approach that targets the parasite hemoglobin receptor.

Leishmaniasis is a vector-borne disease caused by obligate, protozoan parasites of the genus *Leishmania*. These parasites are transmitted by 30 different species of *Phlebotomine* sand flies as extracellular, flagellated promastigotes and replicate as intracellular, aflagellate amastigotes in mononuclear phagocytes in mammalian hosts.^1, 2^ Leishmaniasis ranges from self healing, asymptomatic infection to localized skin lesions, and can develop into a life-threatening progressive visceral form of disease. Leishmaniasis is one of the world's most neglected diseases, affecting mainly very poor people in developing countries. It is prevalent throughout the tropical and subtropical regions of Africa, Asia, the Mediterranean, Southern Europe (old world) and South and Central America (new world). The disease is endemic in 88 countries, of which 72 are developing countries. Approximately 350 million people are at risk of contracting leishmaniasis and 1.5–2 million new cases occur annually.^3^ The transmission of *Leishmania* parasites is anthroponotic (human to vector to human) in the Indian subcontinent and Asia, while in Africa, Europe and the Americas', it is zoonotic (animal to vector to human), where dogs and rodents act as reservoir (Figure 1).^4, 5, 6^

Visceral leishmaniasis (VL), commonly known as kala-azar, is caused by *L. donovani* and *L. infantum* in the Old World and *L. chagasi* in the New World. These parasites preferentially infect macrophages throughout the viscera, and parasites are usually found in the spleen, liver and bone marrow. Clinical features typically include long-term, low-grade fever, hepatosplenomegaly, weight loss, pancytopenia and polyclonal (IgG and IgM) hypergammaglobulinemia.^7^ Untreated VL will in most cases ultimately lead to death. Approximately 500 000 new cases of VL occur annually.^7^ Most (90%) VL cases occur in five countries, namely; India, Nepal, Bangladesh, Sudan and Brazil.^3^ India is one of the most important foci in the world for VL.^3, 8^ The state of Bihar and neighboring areas of eastern Uttar Pradesh and West Bengal remain particularly badly affected by VL.^9^ The annual incidence of VL in India is approximately 100 000 cases, and the state of Bihar accounts for more than 90% of these.^9^ The incidence of VL-human immunodeficiency virusHIV coinfection is now also a serious concern in these endemic regions.^10^

Cutaneous leishmaniasis (CL) is caused by *L. tropica*, *L. aethiopica* and *L. major* in the Old world, and by *L. mexicana*, *L. guyanensis*, *L. amazonensis* and *L. braziliensis* in the New world. CL is the most common form of leishmaniasis worldwide, representing 50–75% of all new cases. It can be very difficult to treat, long-lasting and disfiguring. According to the World Health Organization (WHO), the number of CL cases is around 1–1.5 million annually, and 90% of CL cases occur in seven countries; Afghanistan, Algeria, Brazil, Iran, Peru, Saudi Arabia and Syria.^3, 6^ CL is characterized by the development of an ulcerative skin lesion, which contains numerous parasites. Although the clinical features of CL can vary because of different causative species, a classical lesion starts as a papule or nodule at the site of parasite inoculation and slowly expands.^6^

Mucocutaneous leishmaniasis (MCL) occurs in the New World and is mainly caused by *L. braziliensis* and *L. panamensis*. These species can metastasize to mucosal tissue in the mouth and upper respiratory tract by lymphatic or haematogenous dissemination, and 90% of all MCL cases occur in Bolivia, Brazil and Peru. MCL can present from several months to years after the development of a cutaneous lesion. Although the pathogenesis of VL and CL is relatively well understood, the pathogenesis of MCL is still unclear, although there has been some recent headway in this area. It is believed that host genetic factors are important for the development of disease.

Control measures for leishmaniasis are heavily dependent on chemotherapy. Currently employed drugs are associated with severe toxic side effects and increasing parasite drug resistance.^11, 12^ This has forced researchers to think about other control measures, and in particular, the development and implementation of an effective vaccine. People cured of *Leishmania* infections develop lifelong immunity. Therefore, prevention of leishmaniasis through prophylactic vaccination is feasible. Advances in our understanding of *Leishmania* infection pathogenesis and the generation of host-protective immunity, together with completed *Leishmania* genome sequences, has opened new avenues for vaccine research. However, major challenges remain, including the translation of ideas from animal models to clinical settings, and the transition of products from the laboratory to the field. This review will highlight recent advancements in the development of vaccines to prevent and/or treat leishmaniasis, and discuss future prospects.

## Protective immune responses in the host

A good understanding of immunity generated against pathogens is important for developing an effective vaccine. Our current understanding of host immune responses generated against *Leishmania* parasites is mainly based on the studies in animal models. Studies in mice show that protective immunity to *Leishmania* infection requires the development of interleukin-12-dependent, parasite-specific Th1 responses, characterized by interferon-γ and tumor necrosis factor production by CD4^+^ T cells.^13, 14, 15^ These inflammatory cytokines are required for the generation of reactive oxygen and nitrogen species by infected macrophages that enables killing of intracellular parasites. Recent advances have also been made in understanding immunoregulatory mechanisms that suppress parasite-specific CD4^+^ T-cell responses in human VL patients. These include the discovery that interleukin-10 produced by CD4^+^ T cells is a potent, autocrine inhibitor of interferon-γ production and promotes parasite persistence in spleen tissue from VL patients.^16^ Thus, interleukin-10 has been identified as a potential therapeutic target for use in combination with drug therapy or to improve therapeutic vaccine efficacy.

The generation of immunological memory is a requirement of effective vaccination. Studies on the generation of effector and central memory CD4^+^ T cells indicate that central memory T cells mediate long-term immunity to *L. major* infection, even in the absence of persistent parasites.^17^ Thus, defining the requirements and understanding the conditions for central memory CD4^+^ T-cell formation and maintenance will be helpful in vaccine design. Our knowledge that the majority of individuals infected with *Leishmania* parasites control parasite growth without causing serious disease,^18, 19^ combined with our understanding about the types of immune responses required for killing parasites and those that suppress this immunity, means that developing vaccines against leishmaniasis is a realistic goal.

## Why do we need a vaccine to prevent and/or treat leishmaniasis?

Treatment of leishmaniasis is dependent on chemotherapy. The most commonly used drugs are pentavalent antimonials, oral miltefosine, amphotericin B, liposomal amphotericin B and paramomycin. A major problem is that these drugs are associated with problems of cost, toxicity, length and duration of treatment, route of injection (for example, intravenous infusion) and the development of parasite drug resistance.^20^ Pentavalent antimonials were the first line of treatment for many years, but increasing parasite resistance in endemic regions has limited their use. In the state of Bihar in India, almost 60% of cases are refractory to treatment with this drug.^21^ Consequently, amphotericin B is now used as the main drug to treat VL patients. However, this drug is also associated with toxicity and there are reports of drug-resistant parasites.^22^ Miltefosine was developed as an oral drug and showed an early promise; however, there are now increasing incidences of relapse in patients treated with this drug.^23, 24, 25^ Recently, a single dose of ambisome (lipid formulation of amphotericin B) was shown to be effective in treating VL patients, with a lower incidence of toxicity, compared with conventional treatment, in a multicentre clinical trial.^26^ Nevertheless, there are concerns that this type of drug-treatment protocol may promote the development of drug-resistant parasites. Therefore, combination drug therapy is being actively developed for use in endemic regions.^27, 28^ However, studies in a mouse model suggest that the *L. donovani* can develop resistance to drugs, even when they are used in combination.^29^ Therefore, despite advances in chemotherapeutic options, it is unlikely that chemotherapy alone will enable disease elimination, and hence there is an urgent need for an effective vaccine if long-term goals to controlling and eliminating this disease are to be achieved.

## Past and present vaccine candidates

Despite different *Leishmania* species causing a range of clinical manifestations, genomic analysis indicates a large degree of sequence homology between species, suggesting it may be possible to generate broadly effective vaccines against different clinical diseases. An effective vaccine against leishmaniasis has existed in the past. This involved inoculation with live, virulent parasites, in a process called leishmanization. It was practiced successfully in the former Soviet Union, Middle East and Israel.^30, 31^ However, it was abandoned in most countries because of logistical problems and safety concerns, due to some individuals developing non-healing lesions and immune suppression.^32^

Whole-killed (autoclaved) *Leishmania* promastigotes were also tested as vaccines against CL and VL. Testing of killed parasite vaccines took place in Brazil in the early 1940s, and was then tested either alone or in combination with adjuvant in phase-I, II and III trials.^33, 34^ Clinical trials with autoclaved *Leishmania,* adjuvanted with BCG, showed that this approach could reduce the incidence of CL by 18–78%.^35, 36^ Similar trials were conducted in Iran, Sudan and Ecuador with variable safety and efficacy.^37, 38, 39, 40, 41^ Unfortunately, the autoclaved parasites showed decreasing potency with time, although studies with thimerosal preserved and non-autoclaved preparations have shown reduced effects of storage.^42^ However, concerns remain regarding the feasibility of developing killed, whole-parasite vaccines, including the variation in results obtained from different field and clinical trial sites in the past, and potential difficulties in producing such a product to good clinical manufacturing standards.

Various attenuated parasites have also been tested in animal models. These parasites are generally taken up by host cells in a similar way to virulent parasites, and persist for some time without replicating. This allows the host to mount robust immune responses against parasite antigens. Radio-attenuated and biochemically altered parasites have proven to confer good protection in mice and hamsters without adjuvant,^43^ although concerns regarding conversion back to virulence make the latter option questionable for human use. However, targeted elimination of virulence genes may overcome this problem and could produce attractive vaccine candidates against leishmaniasis. Genetically modified *Leishmania* parasites lacking essential genes like dyhydrofolate reductase, biopterin reductase or cystein proteases have been shown to stimulate protection against challenge with virulent parasite strains.^44, 45, 46^ The use of drug-sensitive *Leishmania* mutants^47^ alone or with adjuvant has been proposed as a mechanism to induce anti-leishmanial immunity, as has the use of non-pathogenic *Leishmania* species like *L. tarantolae*, which can stimulate protection against virulent *L. donovani* strains.^48^ However, the main problem with using killed or attenuated parasites are the concerns relating to safety and feasibility for large-scale use in the field.

Other approaches include using immunogenic surface antigens of *Leishmania* parasites as vaccine candidates. Several of these have been tested in mouse models and canine VL with data suggesting that protection against leishmaniasis can be achieved with defined candidate proteins. A saponin formulation of fucose mannose ligand that is expressed throughout the life cycle of parasite, was found to be safe, protective and immunogenic in an experimental mouse and hamster models.^49, 50^ This formulation has now become the Leishmune veterinary vaccine, licensed after a series of canine VL field studies.^51, 52^ Lipid formulations of soluble leishmania antigen from *L. donovani* were also tested as vaccine candidates in a hamster model of *L. donovani* infection, and this conferred protection with increased delayed type hypersensitive reactions in response to parasite antigen, enhanced parasite-specific antibody responses and improved parasite-specific T-cell responses.^53, 54^ Liposomal soluble leishmania antigen (from *L. major*) incorporated with phosphorothioate CpG ODN (PS CpG) or phosphodiaster CpG ODN (PO CpG) has also been tested in a mouse model of CL, and generated significant levels of protection.^55^ The excretory/secretory proteins isolated from culture supernatants of *L. infantum* and adjuvanted with muramyl dipeptide were tested in dogs experimentally infected with *L. infantum*.^56^ This vaccine, termed LiESAp-MDP, induced significant, long-lasting protection against canine VL in a field trial in an endemic area of France with naturally infected dogs.^57^ However, a major hurdle with these fractionated vaccines for human applications is their production to good clinical manufacturing standards, as well as gene variation and polymorphisms in field isolates.

Recombinant proteins, either alone or combined with adjuvant or with bacteria/recombinant virus as a delivery vehicles,^58, 59^ have also been tested as vaccines in preclinical studies. There have been significant efforts in recent time to identify recombinant antigens that can protect against *Leishmania* infection in experimental models. Some of these antigens include kinetoplastid membrane protein-11,^60, 61^ sterol 24-c-methyltranferase,^62^ amastigote specific protein A2,^63^ cysteine proteinase B,^64^ *L. braziliensis* elongation and initiation factor,^65^ K26/HASPB,^66^ *Leishmania*-activated C kinase,^67^ promastigote surface antigen 2,^68^ nucleoside hydrolase^69^ and surface expressed glycoprotein gp63.^70^ Although most of these recombinant antigens have been tested in animal models for their immunogenicity and protective efficacy, only a few have progressed to clinical trials in non-human primates, dogs or in preclinical human studies.^71, 72^ A multisubunit recombinant *Leishmania* vaccine, Leish-111F, containing a *L. major* homolog of eukaryotic thiole-specific antioxidant, *L. major* stress inducible protein-1 and *L. braziliensis* elongation and initiation factor, in formulation with MPL-SE, has been shown to provide protection in mouse models of CL and VL,^73, 74^ but failed to prevent canine VL caused by natural *L. infantum* infection.^75^ Nevertheless, Leish-111F/MPL-SE is the first defined vaccine candidate to progress to human phase-I and phase-II clinical trials in healthy volunteers in South America, CL and ML patients in Brazil and Peru and patients cured of VL in India.^76, 77, 78, 79^ As with all subunit vaccines, potential problems include variations in immunogenicity, based on human lymphocyte antigen expression in individuals, gene variation and polymorphisms in parasites, as well as the potential to drive selective pressure of parasites away from the molecules used in vaccines.

Finally, DNA vaccines to prevent leishmaniasis are also undergoing development and testing. This approach is not new,^80^ but has several advantages, such as low costs of production, stability of materials, sustained expression of relevant antigens and efficient generation of effector and memory immune responses.^81^ In addition, more than one antigen can be produced by a single construct. The non-methylated CpG motif of bacterial DNA provides the further advantage of activating innate immune cells to produce interleukin-12, which can prime CD4^+^ T cells to develop into Th1 cells.^82^ A list of vaccine antigen candidates being tested in DNA vaccines for CL and VL is shown in Table 1.

Therefore, despite many years of effort in identifying immunogenic parasite antigens and advances in vaccine technologies, there does not yet appear to be a vaccine candidate capable of delivering the level of protection needed for a disease elimination program. However, a significant advance was recently described in a study by Guha *et al.*,^83^ where they targeted the parasite hemoglobin receptor (HbR) using a DNA vaccine approach and tested it in an experimental model of VL. *Leishmania* parasites require heme for various metabolic activities; however, they lack an endogenous heme synthesis pathway, thus making them dependent on the host. HbR is expressed on the cell surface of the parasite and is conserved among different species. This receptor is not only important for hemoglobin endocytosis,^84^ but also has hexokinase activity, distinct from host hexokinase,^85^ which is important for regulating glycolysis. These important properties of parasite HbR led Guha and colleagues to test this molecule as a DNA vaccine candidate.

**Figure 2:**
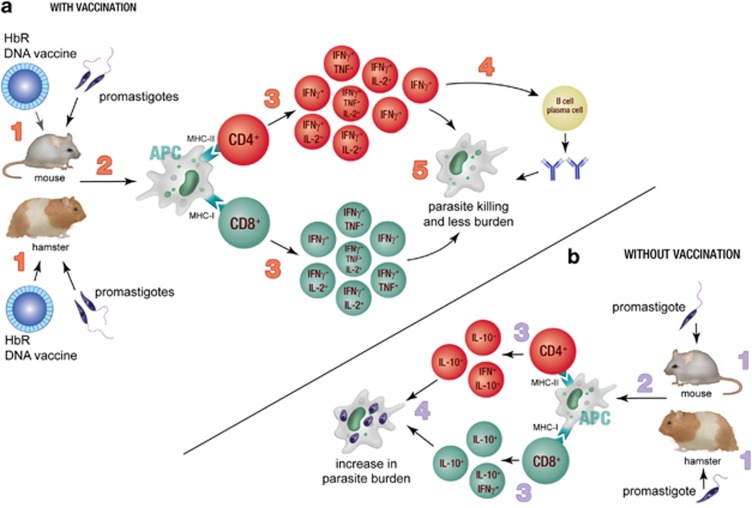
The effect of HbR-DNA vaccination. (**a**) Mice and hamsters were vaccinated with the HbR-DNA vaccine before infection with *L. donovani* promastigotes (1). The antigens were presented by antigen-presenting cells (APC) to CD4^+^ and CD8^+^ T cells in context of MHC-II and MHC-I molecules, respectively (2). This led to the enhanced proliferation of both CD4^+^ and CD8^+^ T cells (3) and generation of multifunctional CD4^+^ and CD8^+^ T cells. CD4^+^ T cells enhanced antibody generation by B cells/plasma cells (4). The combined effect of increased multifunctional CD4^+^ and CD8^+^ T cells and antibody resulted in complete clearance of the parasite (5). (**b**) Non-vaccinated mice and hamsters were infected with *L. donovani* promastigotes (1). Antigens were presented by APC to CD4^+^ and CD8^+^ T cells, (2) which resulted in limited T-cell proliferation and the generation of interleukin-10 producing cells (3) leading to enhanced parasite growth (4).

The success of any vaccine depends on many factors, including the generation of effective antigen-specific antibody responses, the priming and maintenance of parasite-specific T-cell responses and the generation of T cells with appropriate effector functions. Guha *et al.* found that patients with active VL produced reactive antibodies against HbR, and that these antibodies were able to inhibit parasite growth in a complement dependent manner *in vitro*. They also showed that HbR-DNA vaccination of mice stimulated the production of antigen-specific IgG2a antibodies and promoted the generation of antigen-specific T-cell responses that were able to produce multiple Th1-related cytokines simultaneously (that is, a polyfunctional T-cell response). Moreover, immunization with this DNA vaccine enabled sterile cure in hamsters and mice following challenge with virulent *L. donovani* (Figure 2). This is remarkable. These results were obtained in the absence of adjuvant, and thus highlight the potential of HbR as a DNA vaccine candidate for human use. However, further testing, including independent validation of efficacy, must be performed. In addition, and as mentioned earlier, DNA vaccines have shown great promise in animal models, but have not yet proven their utility in humans. There have been no clinical trials beyond phase-II to test DNA vaccines in humans. Thus, a major challenge for DNA vaccine candidates, such as parasite HbR, remains the demonstration of safety and efficacy in humans in both clinical trial and field settings.

## Concluding remarks: problems and future direction

Vaccination is the most cost-effective way of controlling infectious diseases. The success of vaccine development depends upon understanding the immunobiology of pathogen/host interactions, selection of appropriate vaccine candidates and choosing the right adjuvant or delivery vehicle. In addition, the vaccine must be able to generate long-lasting immunity, the best immune correlates of protection must be identified so vaccine efficacy can be efficiently evaluated and it must be able to transition from preclinical testing to human trials. However, despite a better understanding of immune regulatory pathways established following infection or vaccination, we still have a limited capacity to modulate these to clinical advantage with available adjuvants or drugs. Ideally, the vaccine should also be effective against all causative agents for a particular disease. This would allow significant saving in product development and testing, which will be an important consideration in future vaccine development programs. The development of a vaccine against leishmaniasis has been slow. However, increased knowledge gained in recent years in all of the above areas is paving the way for renewed efforts to make and test new vaccines aimed at preventing and/or treating leishmaniasis. If funding sources can be identified and commit to the long road of vaccine development, we are confident this is one parasitic disease that can ultimately be controlled.

## Figures and Tables

**Figure 1:**
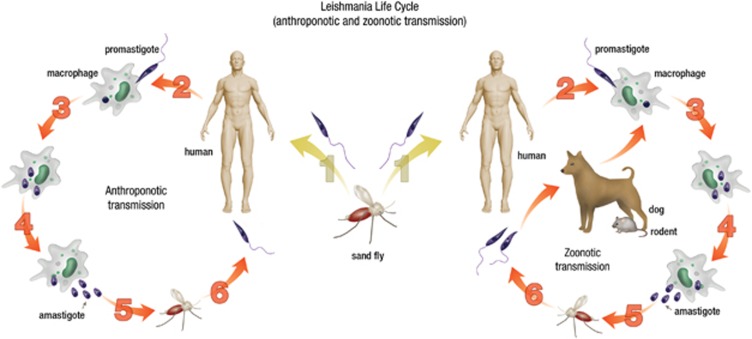
Life cycle and transmission of *Leishmania* parasites. The promastigote form of *Leishmania* parasites responsible for human disease (VL, CL and MCL) are injected into the skin as a female sand fly takes a blood meal (1), and are then taken up by host macrophages (2). Promastigotes convert to the non-flagellated, amastigote form inside macrophages (3) and then divide by binary fission (4). The amastigotes are released by the rupture of macrophages (5) and can then be taken up by a female sand fly during another blood meal. The amastigote form then converts in the promastigote form in the midgut of the sand fly and can then again be transmitted to another human (anthroponotic transmission) or to animals that act as reservoirs (zoonotic transmission) (6).

**Table 1:** *Leishmania* vaccine antigens being tested as candidate DNA vaccines

*Candidate antigen*	*Models*	*Disease*	*Species*	*Reference*
LACK	Dog, mice	VL, CL	*L. donovani, L. chagasi, L. major, L. infantum*	^86, 87, 88, 89, 90^
gp63	Mice, dogs	CL, VL	*L. major, L. infantum*	^88, 90^
KMP11	Mice	CL, VL	*L. major L. donovani*	^91, 92, 93^
CPB	Dogs, mice	VL, CL	*L. infantum, L. major*	^94, 95^
ORFF	Mice	VL	*L. donovani*	^96^
NH 36	Mice	VL, CL	*L. chagasi, L. maxicana*	^97, 98^
TRYP	Dogs	VL	*L. infantum*	^90^
PSA-2	Mice	CL	*L. major*	^88^

Abbreviations: CPB, cysteine proteinase B; CL, cutaneous leishmaniasis; gp63, glycoprotein 63; KMP11, kinetoplastid membrane protein-11; LACK, *Leishmania*-activated C kinase; NH36, nucleoside hydrolase 36; ORFF, open reading frame F; PSA-2, promastigote surface antigen 2; TRYP, tryparedoxin peroxidase; VL, visceral leishmaniasis.

## References

[bib1] Pearson RD Sousa AQ Clinical spectrum of Leishmaniasis Clin Infect Dis 1996 22 1 13 8824958 10.1093/clinids/22.1.1

[bib2] Sacks D Kamhawi S Molecular aspects of parasite-vector and vector–host interactions in leishmaniasis Annu Rev Microbiol 2001 55 453 483 11544364 10.1146/annurev.micro.55.1.453

[bib3] Alvar J Velez ID Bern C Herrero M Desjeux P Cano J Leishmaniasis worldwide and global estimates of its incidence PLoS One 2012 7 e35671 22693548 10.1371/journal.pone.0035671 PMC3365071

[bib4] Alvar J Canavate C Molina R Moreno J Nieto J Canine leishmaniasis Advances in parasitology 2004 57 1 88 15504537 10.1016/S0065-308X(04)57001-X

[bib5] Postigo JA Leishmaniasis in the World Health Organization Eastern Mediterranean Region I J Antimicrob Agents 2010 36 (Suppl 1 S62 S65 10.1016/j.ijantimicag.2010.06.023 20728317

[bib6] Reithinger R Dujardin JC Louzir H Pirmez C Alexander B Brooker S Cutaneous leishmaniasis Lancet Infect Dis 2007 7 581 596 17714672 10.1016/S1473-3099(07)70209-8

[bib7] Chappuis F Sundar S Hailu A Ghalib H Rijal S Peeling RW Visceral leishmaniasis: what are the needs for diagnosis, treatment and control Nat Rev Microbiol 2007 5 873 882 17938629 10.1038/nrmicro1748

[bib8] Desjeux P Leishmaniasis: current situation and new perspectives Comp Immunol Microbiol Infect Dis 2004 27 305 318 15225981 10.1016/j.cimid.2004.03.004

[bib9] Bora D Epidemiology of visceral leishmaniasis in India Natl Med J India 1999 12 62 68 10416321

[bib10] Desjeux P Global control and leishmania HIV co-infection Clin Dermatol 1999 17 317 325 10384871 10.1016/s0738-081x(99)00050-4

[bib11] Croft SL Sundar S Fairlamb AH Drug resistance in leishmaniasis Clin Microbiol Rev 2006 19 111 126 16418526 10.1128/CMR.19.1.111-126.2006 PMC1360270

[bib12] Sundar S Drug resistance in Indian visceral leishmaniasis Trop Med Int Health 2001 6 849 854 11703838 10.1046/j.1365-3156.2001.00778.x

[bib13] Engwerda CR Ato M Stager S Alexander CE Stanley AC Kaye PM Distinct roles for lymphotoxin-alpha and tumor necrosis factor in the control of *Leishmania donovani* infection Am J Pathol 2004 165 2123 2133 15579454 10.1016/s0002-9440(10)63262-2 PMC1618729

[bib14] Solbach W Laskay T The host response to *Leishmania* infection Adv Immunol 2000 74 275 317 10605608 10.1016/s0065-2776(08)60912-8

[bib15] Squires KE Schreiber RD McElrath MJ Rubin BY Anderson SL Murray HW Experimental visceral leishmaniasis: role of endogenous IFN-gamma in host defense and tissue granulomatous response J Immunol 1989 143 4244 4249 2512353

[bib16] Gautam S Kumar R Maurya R Nylen S Ansari N Rai M IL-10 neutralization promotes parasite clearance in splenic aspirate cells from patients with visceral leishmaniasis J Infect Dis 2011 204 1134 1137 21881130 10.1093/infdis/jir461 PMC3164427

[bib17] Zaph C Uzonna J Beverley SM Scott P Central memory T cells mediate long-term immunity to *Leishmania major* in the absence of persistent parasites Nat Med 2004 10 1104 1110 15448686 10.1038/nm1108

[bib18] Badaro R Jones TC Carvalho EM Sampaio D Reed SG Barral A New perspectives on a subclinical form of visceral leishmaniasis J Infect Dis 1986 154 1003 1011 3782864 10.1093/infdis/154.6.1003

[bib19] Zijlstra EE el-Hassan AM Ismael A Ghalib HW Endemic kala-azar in eastern Sudan: a longitudinal study on the incidence of clinical and subclinical infection and post-kala-azar dermal leishmaniasis Am J Trop Med Hyg 1994 51 826 836 7810819 10.4269/ajtmh.1994.51.826

[bib20] Chakravarty J Sundar S Drug resistance in leishmaniasis J Glob Infect Dis 2010 2 167 176 20606973 10.4103/0974-777X.62887 PMC2889657

[bib21] Agrawal S Rai M Sundar S Management of visceral leishmaniasis: Indian perspective J Postgrad Med 2005 51 (Suppl 1 S53 S57 16519257

[bib22] Srivastava P Prajapati VK Rai M Sundar S Unusual case of resistance to amphotericin B in visceral leishmaniasis in a region in India where leishmaniasis is not endemic J Clin Microbiol 2011 49 3088 3091 21613432 10.1128/JCM.00173-11 PMC3146580

[bib23] Bhandari V Kulshrestha A Deep DK Stark O Prajapati VK Ramesh V Drug susceptibility in *Leishmania* isolates following miltefosine treatment in cases of visceral leishmaniasis and post kala-azar dermal leishmaniasis PLoS Negl Trop Dis 2012 6 e1657 22629478 10.1371/journal.pntd.0001657 PMC3358331

[bib24] Rijal S Ostyn B Uranw S Rai K Bhattarai NR Dorlo TP Increasing failure of miltefosine in the treatment of kala-azar in Nepal and the potential role of parasite drug resistance, reinfection, or noncompliance Clin Infect Dis 2013 56 1530 1538 23425958 10.1093/cid/cit102

[bib25] Sundar S Singh A What steps can be taken to counter the increasing failure of miltefosine to treat visceral leishmaniasis Expert Rev Anti Infect Ther 2013 11 117 119 23409817 10.1586/eri.12.170

[bib26] Sundar S Chakravarty J Agarwal D Rai M Murray HW Single-dose liposomal amphotericin B for visceral leishmaniasis in India N Engl J Med 2010 362 504 512 20147716 10.1056/NEJMoa0903627

[bib27] Sundar S Sinha PK Rai M Verma DK Nawin K Alam S Comparison of short-course multidrug treatment with standard therapy for visceral leishmaniasis in India: an open-label, non-inferiority, randomised controlled trial Lancet 2011 377 477 486 21255828 10.1016/S0140-6736(10)62050-8

[bib28] Sundar S Chakravarty J Leishmaniasis: an update of current pharmacotherapy Expert Opin Pharmacother 2013 14 53 63 23256501 10.1517/14656566.2013.755515

[bib29] Garcia-Hernandez R Manzano JI Castanys S Gamarro F Leishmania donovani develops resistance to drug combinations PLoS Negl Trop Dis 2012 6 e1974 23285310 10.1371/journal.pntd.0001974 PMC3527373

[bib30] Greenblatt CL The present and future of vaccination for cutaneous leishmaniasis Prog Clin Biol Res 1980 47 259 285 7010374

[bib31] Handman E Leishmaniasis: current status of vaccine development Clin Microbiol Rev 2001 14 229 243 11292637 10.1128/CMR.14.2.229-243.2001 PMC88972

[bib32] Nadim A Javadian E Tahvildar-Bidruni G Ghorbani M Effectiveness of leishmanization in the control of cutaneous leishmaniasis Bull Soc Pathol Exot Filiales 1983 76 377 383 6354498

[bib33] Momeni AZ Jalayer T Emamjomeh M Khamesipour A Zicker F Ghassemi RL A randomised, double-blind, controlled trial of a killed *L. major* vaccine plus BCG against zoonotic cutaneous leishmaniasis in Iran Vaccine 1999 17 (5 466 472 10073725 10.1016/s0264-410x(98)00220-5

[bib34] Velez ID del Pilar Agudelo S Arbelaez MP Gilchrist K Robledo SM Puerta JA Safety and immunogenicity of a killed *Leishmania (L.) amazonensis* vaccine against cutaneous leishmaniasis in Colombia: a randomized controlled trial Trans R Soc Trop Med Hyg 2000 94 698 703 11198661 10.1016/s0035-9203(00)90239-6

[bib35] Antunes CM Mayrink W Magalhaes PA Costa CA Melo MN Dias M Controlled field trials of a vaccine against New World cutaneous leishmaniasis Int J Epidemiol 1986 15 572 580 3546170 10.1093/ije/15.4.572

[bib36] Sharifi I FeKri AR Aflatonian MR Khamesipour A Nadim A Mousavi MR Randomised vaccine trial of single dose of killed *Leishmania major* plus BCG against anthroponotic cutaneous leishmaniasis in Bam, Iran Lancet 1998 351 1540 1543 10326536 10.1016/S0140-6736(98)09552-X

[bib37] Dowlati Y Ehsasi S Shidani B Bahar K Stepwise safety trial of a killed *Leishmania* vaccine in Iran Clin Dermatol 1996 14 497 502 8889327 10.1016/0738-081x(96)00072-7

[bib38] Bahar K Dowlati Y Shidani B Alimohammadian MH Khamesipour A Ehsasi S Comparative safety and immunogenicity trial of two killed *Leishmania major* vaccines with or without BCG in human volunteers Clin Dermatol 1996 14 489 495 8889326 10.1016/0738-081x(96)00071-5

[bib39] Armijos RX Weigel MM Aviles H Maldonado R Racines J Field trial of a vaccine against New World cutaneous leishmaniasis in an at-risk child population: safety, immunogenicity, and efficacy during the first 12 months of follow-up J Infect Dis 1998 177 1352 1357 9593024 10.1086/515265

[bib40] Khalil EA El Hassan AM Zijlstra EE Mukhtar MM Ghalib HW Musa B Autoclaved *Leishmania major* vaccine for prevention of visceral leishmaniasis: a randomised, double-blind, BCG-controlled trial in Sudan Lancet 2000 356 1565 1569 11075771 10.1016/s0140-6736(00)03128-7

[bib41] Kamil AA Khalil EA Musa AM Modabber F Mukhtar MM Ibrahim ME Alum-precipitated autoclaved *Leishmania major* plus bacille Calmette-Guerrin, a candidate vaccine for visceral leishmaniasis: safety, skin-delayed type hypersensitivity response and dose finding in healthy volunteers Trans R Soc Trop Med Hyg 2003 97 365 368 15228261 10.1016/s0035-9203(03)90171-4

[bib42] De Luca PM Mayrink W Alves CR Coutinho SG Oliveira MP Bertho AL Evaluation of the stability and immunogenicity of autoclaved and nonautoclaved preparations of a vaccine against American tegumentary leishmaniasis Vaccine 1999 17 1179 1185 10195630 10.1016/s0264-410x(98)00338-7

[bib43] Rivier D Bovay P Shah R Didisheim S Mauel J Vaccination against Leishmania major in a CBA mouse model of infection: role of adjuvants and mechanism of protection Parasite Immunol 1999 21 461 473 10476055 10.1046/j.1365-3024.1999.00244.x

[bib44] Souza AE Bates PA Coombs GH Mottram JC Null mutants for the lmcpa cysteine proteinase gene in *Leishmania mexicana* Mol Biochem Parasitol 1994 63 213 220 8008019 10.1016/0166-6851(94)90057-4

[bib45] El Fadili A Kundig C Roy G Ouellette M Inactivation of the Leishmania tarentolae pterin transporter (BT1) and reductase (PTR1) genes leads to viable parasites with changes in folate metabolism and hypersensitivity to the antifolate methotrexate J Biol Chem 2004 279 18575 18582 14981076 10.1074/jbc.M400652200

[bib46] Veras P Brodskyn C Balestieri F Freitas L Ramos A Queiroz A A dhfr-ts- *Leishmania major* knockout mutant cross-protects against Leishmania amazonensis Mem Inst Oswaldo Cruz 1999 94 491 496 10446007 10.1590/s0074-02761999000400011

[bib47] Muyombwe A Olivier M Ouellette M Papadopoulou B Selective killing of *Leishmania amastigotes* expressing a thymidine kinase suicide gene Exp Parasitol 1997 85 35 42 9024200 10.1006/expr.1996.4115

[bib48] Breton M Tremblay MJ Ouellette M Papadopoulou B Live nonpathogenic parasitic vector as a candidate vaccine against visceral leishmaniasis Infect Immun 2005 73 6372 6382 16177308 10.1128/IAI.73.10.6372-6382.2005 PMC1230936

[bib49] Palatnik-de-Sousa CB Paraguai-de-Souza E Gomes EM Borojevic R Experimental murine *Leishmania donovani* infection: immunoprotection by the fucose-mannose ligand (FML) Braz J Med Biol Res 1994 27 547 551 8081280

[bib50] Santos WR Aguiar IA Paraguai de Souza E de Lima VM Palatnik M Palatnik-de-Sousa CB Immunotherapy against murine experimental visceral leishmaniasis with the FML-vaccine Vaccine 2003 21 4668 4676 14585674 10.1016/s0264-410x(03)00527-9

[bib51] da Silva VO Borja-Cabrera GP Correia Pontes NN de Souza EP Luz KG Palatnik M A phase III trial of efficacy of the FML-vaccine against canine kala-azar in an endemic area of Brazil (Sao Goncalo do Amaranto, RN) Vaccine 2000 19 1082 1092 11137242 10.1016/s0264-410x(00)00339-x

[bib52] Parra LE Borja-Cabrera GP Santos FN Souza LO Palatnik-de-Sousa CB Menz I Safety trial using the Leishmune vaccine against canine visceral leishmaniasis in Brazil Vaccine 2007 25 2180 2186 17239495 10.1016/j.vaccine.2006.11.057

[bib53] Afrin F Ali N Adjuvanticity and protective immunity elicited by *Leishmania donovani* antigens encapsulated in positively charged liposomes Infect Immun 1997 65 2371 2377 9169776 10.1128/iai.65.6.2371-2377.1997 PMC175328

[bib54] Sharma SK Dube A Nadeem A Khan S Saleem I Garg R Non PC liposome entrapped promastigote antigens elicit parasite specific CD8+ and CD4+ T-cell immune response and protect hamsters against visceral leishmaniasis Vaccine 2006 24 1800 1810 16310900 10.1016/j.vaccine.2005.10.025

[bib55] Shargh VH Jaafari MR Khamesipour A Jaafari I Jalali SA Abbasi A Liposomal SLA co-incorporated with PO CpG ODNs or PS CpG ODNs induce the same protection against the murine model of leishmaniasis Vaccine 2012 30 3957 3964 22465747 10.1016/j.vaccine.2012.03.040

[bib56] Lemesre JL Holzmuller P Cavaleyra M Goncalves RB Hottin G Papierok G Protection against experimental visceral leishmaniasis infection in dogs immunized with purified excreted secreted antigens of *Leishmania infantum* promastigotes Vaccine 2005 23 2825 2840 15780731 10.1016/j.vaccine.2004.11.061

[bib57] Lemesre JL Holzmuller P Goncalves RB Bourdoiseau G Hugnet C Cavaleyra M Long-lasting protection against canine visceral leishmaniasis using the LiESAp-MDP vaccine in endemic areas of France: double-blind randomised efficacy field trial Vaccine 2007 25 4223 4234 17395339 10.1016/j.vaccine.2007.02.083

[bib58] Xu D McSorley SJ Chatfield SN Dougan G Liew FY Protection against *Leishmania major* infection in genetically susceptible BALB/c mice by gp63 delivered orally in attenuated *Salmonella typhimurium* (AroA- AroD-) Immunology 1995 85 1 7 7635511 PMC1384017

[bib59] Maroof A Brown N Smith B Hodgkinson MR Maxwell A Losch FO Therapeutic vaccination with recombinant adenovirus reduces splenic parasite burden in experimental visceral leishmaniasis J Infect Dis 2012 205 853 863 22301630 10.1093/infdis/jir842 PMC3274377

[bib60] Agallou M Margaroni M Karagouni E Cellular vaccination with bone marrow-derived dendritic cells pulsed with a peptide of *Leishmania infantum* KMP-11 and CpG oligonucleotides induces protection in a murine model of visceral leishmaniasis Vaccine 2011 29 5053 5064 21569815 10.1016/j.vaccine.2011.04.089

[bib61] Carrillo E Crusat M Nieto J Chicharro C Thomas Mdel C Martinez E Immunogenicity of HSP-70, KMP-11 and PFR-2 leishmanial antigens in the experimental model of canine visceral leishmaniasis Vaccine 2008 26 1902 1911 18321614 10.1016/j.vaccine.2008.01.042

[bib62] Goto Y Bogatzki LY Bertholet S Coler RN Reed SG Protective immunization against visceral leishmaniasis using Leishmania sterol 24-c-methyltransferase formulated in adjuvant Vaccine 2007 25 7450 7458 17804125 10.1016/j.vaccine.2007.08.001 PMC2077354

[bib63] Ghosh A Zhang WW Matlashewski G Immunization with A2 protein results in a mixed Th1/Th2 and a humoral response which protects mice against *Leishmania donovani* infections Vaccine 2001 20 59 66 11567746 10.1016/s0264-410x(01)00322-x

[bib64] Rafati S Zahedifard F Nazgouee F Prime-boost vaccination using cysteine proteinases type I and II of *Leishmania infantum* confers protective immunity in murine visceral leishmaniasis Vaccine 2006 24 2169 2175 16325969 10.1016/j.vaccine.2005.11.011

[bib65] Skeiky YA Kennedy M Kaufman D Borges MM Guderian JA Scholler JK LeIF: a recombinant Leishmania protein that induces an IL-12-mediated Th1 cytokine profile J Immunol 1998 161 6171 6179 9834103

[bib66] Stager S Smith DF Kaye PM Immunization with a recombinant stage-regulated surface protein from *Leishmania donovani* induces protection against visceral leishmaniasis J Immunol 2000 165 7064 7071 11120835 10.4049/jimmunol.165.12.7064

[bib67] Benhnini F Chenik M Laouini D Louzir H Cazenave PA Dellagi K Comparative evaluation of two vaccine candidates against experimental leishmaniasis due to *Leishmania major* infection in four inbred mouse strains Clin Vaccine Immunol 2009 16 1529 1537 19726616 10.1128/CVI.00153-09 PMC2772381

[bib68] Handman E Symons FM Baldwin TM Curtis JM Scheerlinck JP Protective vaccination with promastigote surface antigen 2 from *Leishmania major* is mediated by a TH1 type of immune response Infect Immun 1995 63 4261 4267 7591056 10.1128/iai.63.11.4261-4267.1995 PMC173605

[bib69] Al-Wabel MA Tonui WK Cui L Martin SK Titus RG Protection of susceptible BALB/c mice from challenge with *Leishmania major* by nucleoside hydrolase, a soluble exo-antigen of *Leishmania* Am J Trop Med Hyg 2007 77 1060 1065 18165522

[bib70] Connell ND Medina-Acosta E McMaster WR Bloom BR Russell DG Effective immunization against cutaneous leishmaniasis with recombinant bacille Calmette-Guerin expressing the Leishmania surface proteinase gp63 Proc Natl Acad Sci USA 1993 90 11473 11477 8265576 10.1073/pnas.90.24.11473 PMC48006

[bib71] Kumar R Goto Y Gidwani K Cowgill KD Sundar S Reed SG Evaluation of *ex vivo* human immune response against candidate antigens for a visceral leishmaniasis vaccine Am J Trop Med Hyg 2010 82 808 813 20439959 10.4269/ajtmh.2010.09-0341 PMC2861380

[bib72] Singh OP Stober CB Singh AK Blackwell JM Sundar S Cytokine responses to novel antigens in an Indian population living in an area endemic for visceral leishmaniasis PLoS Negl Trop Dis 2012 6 e1874 23150744 10.1371/journal.pntd.0001874 PMC3493615

[bib73] Skeiky YA Coler RN Brannon M Stromberg E Greeson K Crane RT Protective efficacy of a tandemly linked, multi-subunit recombinant leishmanial vaccine (Leish-111f) formulated in MPL adjuvant Vaccine 2002 20 3292 3303 12213399 10.1016/s0264-410x(02)00302-x

[bib74] Coler RN Goto Y Bogatzki L Raman V Reed SG Leish-111f, a recombinant polyprotein vaccine that protects against visceral Leishmaniasis by elicitation of CD4+ T cells Infect Immun 2007 75 4648 4654 17606603 10.1128/IAI.00394-07 PMC1951162

[bib75] Gradoni L Foglia Manzillo V Pagano A Piantedosi D De Luna R Gramiccia M Failure of a multi-subunit recombinant leishmanial vaccine (MML) to protect dogs from *Leishmania infantum* infection and to prevent disease progression in infected animals Vaccine 2005 23 5245 5251 16054272 10.1016/j.vaccine.2005.07.001

[bib76] Velez ID Gilchrist K Martinez S Ramirez-Pineda JR Ashman JA Alves FP Safety and immunogenicity of a defined vaccine for the prevention of cutaneous leishmaniasis Vaccine 2009 28 329 337 19879995 10.1016/j.vaccine.2009.10.045

[bib77] Nascimento E Fernandes DF Vieira EP Campos-Neto A Ashman JA Alves FP A clinical trial to evaluate the safety and immunogenicity of the LEISH-F1+MPL-SE vaccine when used in combination with meglumine antimoniate for the treatment of cutaneous leishmaniasis Vaccine 2010 28 6581 6587 20688040 10.1016/j.vaccine.2010.07.063

[bib78] Llanos-Cuentas A Calderon W Cruz M Ashman JA Alves FP Coler RN A clinical trial to evaluate the safety and immunogenicity of the LEISH-F1+MPL-SE vaccine when used in combination with sodium stibogluconate for the treatment of mucosal leishmaniasis Vaccine 2010 28 7427 7435 20851080 10.1016/j.vaccine.2010.08.092

[bib79] Chakravarty J Kumar S Trivedi S Rai VK Singh A Ashman JA A clinical trial to evaluate the safety and immunogenicity of the LEISH-F1+MPL-SE vaccine for use in the prevention of visceral leishmaniasis Vaccine 2011 29 3531 3537 21414377 10.1016/j.vaccine.2011.02.096

[bib80] Tang DC DeVit M Johnston SA Genetic immunization is a simple method for eliciting an immune response Nature 1992 356 152 154 1545867 10.1038/356152a0

[bib81] Donnelly JJ Ulmer JB Shiver JW Liu MA DNA vaccines Annu Rev Immunol 1997 15 617 648 9143702 10.1146/annurev.immunol.15.1.617

[bib82] Gurunathan S Klinman DM Seder RA DNA vaccines: immunology, application, and optimization Annu Rev Immunol 2000 18 927 974 10837079 10.1146/annurev.immunol.18.1.927

[bib83] Guha R Gupta D Rastogi R Vikram R Krishnamurthy G Bimal S Vaccination with leishmania hemoglobin receptor-encoding DNA protects against visceral leishmaniasis Sci Transl Med 2013 5 202ra121 10.1126/scitranslmed.3006406 24027025

[bib84] Sengupta S Tripathi J Tandon R Raje M Roy RP Basu SK Hemoglobin endocytosis in *Leishmania* is mediated through a 46-kDa protein located in the flagellar pocket J Biol Chem 1999 274 2758 2765 9915807 10.1074/jbc.274.5.2758

[bib85] Krishnamurthy G Vikram R Singh SB Patel N Agarwal S Mukhopadhyay G Hemoglobin receptor in *Leishmania* is a hexokinase located in the flagellar pocket J Biol Chem 2005 280 5884 5891 15579464 10.1074/jbc.M411845200

[bib86] Melby PC Yang J Zhao W Perez LE Cheng J *Leishmania donovani* p36(LACK) DNA vaccine is highly immunogenic but not protective against experimental visceral leishmaniasis Infect Immun 2001 69 4719 4725 11447143 10.1128/IAI.69.8.4719-4725.2001 PMC98557

[bib87] Ramiro MJ Zarate JJ Hanke T Rodriguez D Rodriguez JR Esteban M Protection in dogs against visceral leishmaniasis caused by *Leishmania infantum* is achieved by immunization with a heterologous prime-boost regime using DNA and vaccinia recombinant vectors expressing LACK Vaccine 2003 21 2474 2484 12744881 10.1016/s0264-410x(03)00032-x

[bib88] Ahmed SB Bahloul C Robbana C Askri S Dellagi K A comparative evaluation of different DNA vaccine candidates against experimental murine leishmaniasis due to *L. major* Vaccine 2004 22 1631 1639 15068845 10.1016/j.vaccine.2003.10.046

[bib89] Marques-da-Silva EA Coelho EA Gomes DC Vilela MC Masioli CZ Tavares CA Intramuscular immunization with p36(LACK) DNA vaccine induces IFN-gamma production but does not protect BALB/c mice against *Leishmania chagasi* intravenous challenge Parasitol Res 2005 98 67 74 16261353 10.1007/s00436-005-0008-8

[bib90] Rodriguez-Cortes A Ojeda A Lopez-Fuertes L Timon M Altet L Solano-Gallego L Vaccination with plasmid DNA encoding KMPII, TRYP, LACK and GP63 does not protect dogs against *Leishmania infantum* experimental challenge Vaccine 2007 25 7962 7971 17942199 10.1016/j.vaccine.2007.08.023

[bib91] Basu R Bhaumik S Basu JM Naskar K De T Roy S Kinetoplastid membrane protein-11 DNA vaccination induces complete protection against both pentavalent antimonial-sensitive and -resistant strains of *Leishmania donovani* that correlates with inducible nitric oxide synthase activity and IL-4 generation: evidence for mixed Th1- and Th2-like responses in visceral leishmaniasis J Immunol 2005 174 7160 7171 15905560 10.4049/jimmunol.174.11.7160

[bib92] Bhaumik S Basu R Sen S Naskar K Roy S KMP-11 DNA immunization significantly protects against *L. donovani* infection but requires exogenous IL-12as an adjuvant for comparable protection against *L. major* Vaccine 2009 27 1306 1316 19162111 10.1016/j.vaccine.2008.12.053

[bib93] Guha R Das S Ghosh J Naskar K Mandala A Sundar S Heterologous priming-boosting with DNA and vaccinia virus expressing kinetoplastid membrane protein-11 induces potent cellular immune response and confers protection against infection with antimony resistant and sensitive strains of *Leishmania* (*Leishmania*) *donovani* Vaccine 2013 31 1905 1915 23499564 10.1016/j.vaccine.2013.02.025

[bib94] Rafati S Salmanian AH Taheri T Vafa M Fasel N A protective cocktail vaccine against murine cutaneous leishmaniasis with DNA encoding cysteine proteinases of *Leishmania major* Vaccine 2001 19 3369 3375 11348700 10.1016/s0264-410x(01)00081-0

[bib95] Rafati S Nakhaee A Taheri T Taslimi Y Darabi H Eravani D Protective vaccination against experimental canine visceral leishmaniasis using a combination of DNA and protein immunization with cysteine proteinases type I and II of *L. infantum* Vaccine 2005 23 3716 3725 15882533 10.1016/j.vaccine.2005.02.009

[bib96] Sharma A Madhubala R Ubiquitin conjugation of open reading frame F DNA vaccine leads to enhanced cell-mediated immune response and induces protection against both antimony-susceptible and -resistant strains of *Leishmania donovani* J Immunol 2009 183 7719 7731 19933862 10.4049/jimmunol.0900132

[bib97] Gamboa-Leon R Paraguai de Souza E Borja-Cabrera GP Santos FN Myashiro LM Pinheiro RO Immunotherapy against visceral leishmaniasis with the nucleoside hydrolase-DNA vaccine of *Leishmania donovani* Vaccine 2006 24 4863 4873 16635538 10.1016/j.vaccine.2006.03.005

[bib98] Aguilar-Be I da Silva Zardo R Paraguai de Souza E Borja-Cabrera GP Rosado-Vallado M Mut-Martin M Cross-protective efficacy of a prophylactic *Leishmania donovani* DNA vaccine against visceral and cutaneous murine leishmaniasis Infect Immun 2005 73 812 819 15664920 10.1128/IAI.73.2.812-819.2005 PMC547025

